# Comparison of Uncemented and Hybrid Hip Arthroplasty: Protocol for a Brazilian Randomized Controlled Trial

**DOI:** 10.2196/79721

**Published:** 2026-03-16

**Authors:** Bruno Gonçalves Schroder e Souza, Isabella Dias Lacerda, Matheus Malta Vasconcelos, Nathália Lacerda Furtado, Julia Machado Vieira, Iasmim Sand Ferreira de Souza, Marco Antônio Perígolo Magalhães, Leonã Aparecido Homem do Amaral, Luiz Guilherme Vidal Assad, Carlos Henrique Cavaglieri Silveira Silva, Carolina Tsen, Valdeci Manoel de Oliveira

**Affiliations:** 1 Faculdade de Ciências Médicas e da Saúde de Juiz de Fora – Suprema, Alameda Salvaterra, 200, Juiz de Fora, 36033-003, Brazil, 55 (32) 3211-0012; 2 Hospital e Maternidade Therezinha de Jesus, Juiz de Fora, Brazil; 3 Universidade Federal de São Carlos, São Carlos, Brazil

**Keywords:** hip, osteoarthritis, surgical procedures, operative, arthroplasty, replacement, randomized controlled trial

## Abstract

**Background:**

Total hip arthroplasty is a highly successful procedure for treating hip arthritis, improving patients’ pain, function, and quality of life (QoL). Scarce publications on total hip arthroplasty performance using Brazilian-manufactured materials report results comparable to those from other countries.

**Objective:**

The aim of this study is to compare the clinical, radiographic, functional, pain, and QoL outcomes of patients who underwent surgery with a national hybrid prosthesis (with femoral cementation) versus patients who underwent surgery with a national uncemented prosthesis.

**Methods:**

This study is a single-center, single-surgeon, single-approach, pragmatic, double-blinded, and prospective randomized trial. A total of 120 patients will be enrolled and randomly allocated in a 1:1 ratio to 2 groups: the hybrid group and the uncemented group. The incidence of complications (during and after surgery) and the restoration of normal anatomical parameters on postoperative radiographs will be assessed using radiographic parameters. Participants’ QoL, joint mobility, function, and satisfaction will be evaluated using the 12-Item Short Form Health Survey version 2 questionnaire, hip range of motion, the Harris Hip Score, and a numeric rating scale. Prosthesis survival will be analyzed using the annual revision surgery rate from 1 to 5 years, and up to 10 years.

**Results:**

The project did not receive external funding. Data collection began in October 2024 and is ongoing, with completion expected in December 2029. As of April 2025, 72 participants have been enrolled. Preliminary data analysis has been initiated and is ongoing. The first results are expected to be published in the first half of 2026.

**Conclusions:**

This study is a pragmatic clinical trial that uses blinding to evaluate national implants for both hybrid and uncemented hip arthroplasty. Successful completion of this study may provide clinical evidence on the performance of national implants and identify a preferred implant construct (hybrid or uncemented), if any, for hip replacement in Brazil.

## Introduction

Total hip arthroplasty (THA) is a highly successful procedure for treating hip arthritis, improving patients’ pain, function, and quality of life (QoL) [1,2]. However, THA is still vulnerable to complications such as fractures, loosening, infection, as well as other biological issues [3]. Because patients who undergo THA face the risk of cumbersome and costly revision procedures, the life expectancy and durability of the implant are important issues [3]. Different surgical techniques and implants, such as cemented and uncemented stems, are used to perform THA [4]. However, despite the good results of this procedure, no single surgical technique or type of implant has been proven superior for all patients [4].

To date, considerable controversy persists regarding the best type of surgical implant. While in North America, there is a preference for noncemented implants [4], this is mainly based on the findings of a 17-year follow-up randomized controlled trial (RCT) published by Corten et al [5] in 2011. In other countries, especially in Europe, the preference for cemented femoral implants has been maintained. The latter is supported by at least 6 RCTs that show no differences between these types of implants [4], as well as reports of a greater incidence of surgical complications with uncemented implants, especially in older patients [6].

Worldwide, the increasing use of uncemented implants has been described as the “paradox of the decline of cemented implants” [7]. In fact, Troelsen et al [8] in 2013 analyzed 7 national registries, including Australia, Canada, England, Denmark, New Zealand, Norway, and Sweden, in which the use of uncemented prostheses showed a steady increase in the use of this kind of prostheses [8]. Recent studies have highlighted an increased “cementless” trend in some countries between 2010 and 2020; however, this trend appeared to be reversing in others, such as England and Wales, Australia, New Zealand, and Finland [9]. Those authors agree that survival was significantly better for cemented prostheses in all registers in people aged older than 70 to 75 years [8,9]. For other age groups, survival was better for cemented prostheses or equivalent, except in the Australian register. Using another approach to the problem, a meta-analysis including 27 studies conducted by Phedy et al [10] reported very similar results, showing equivalence of both types of implants in the younger patients and superiority of cemented fixation in older patients.

While most RCTs in this area of research have traditionally focused on implant survival and revision rates [4], patient-centered outcomes, QoL, and early complications have become a current hot topic. Hybrid THA yields similar short- to mid-term patient-reported outcome measures (Harris Hip Score [HHS], WOMAC, EQ-5D) compared with cemented and uncemented constructs. A 2025 systematic review of 357,748 THAs found no difference in patient-reported outcome measures between hybrid and cemented THA and no clear advantage of uncemented constructs [11]. However, the incidence of periprosthetic fracture was reported to be significantly higher with uncemented stems, especially in older adults. Moreover, a slightly higher incidence of dislocations was also reported for uncemented stems [12]. Regarding pain, direct elective THA comparisons are limited, but evidence consistently shows more early pain and slower recovery with uncemented femoral fixation due to press-fit stem insertion forces. A meta-analysis of RCTs in THA showed better short-term pain scores with cemented fixation [13]. Additionally, a large National Health Service cost-effectiveness analysis (n≈300,000) found higher postoperative EQ-5D scores with hybrid THA (0.81 vs 0.80 for uncemented), and for most patient groups, hybrid prostheses were found to be the most cost-effective [14].

Few publications on THA performance using Brazilian-manufactured materials have reported results comparable to those from other countries; however, the level of evidence and sample sizes of these studies are limited [15-17]. Additionally, early failures of previous national models, commonly used in Brazil until the 1990s, became the reason for persistent questioning about the safety and durability of Brazilian-made implants [18]. To date, no level 1 study evaluating the performance of such implants in Brazil has been identified.

This study aims to compare the clinical outcomes (function, pain, complications, QoL, and need for revisions) and radiographic outcomes of patients treated with a national hybrid prosthesis (with femoral cementation and uncemented acetabular cup) versus those receiving national uncemented prostheses of the same brand.

## Methods

### Study Design and Setting

This study was written according to the Standard Protocol Items: Recommendations for Interventional Trials (SPIRIT) guidelines (Checklist 1) for protocols of randomized clinical trials. The proposal is a single-center, single-surgeon, single-approach, pragmatic, double-blinded, prospective randomized trial to analyze the differences between uncemented hip replacement and the hybrid (femoral cemented) hip replacement.

Two different types of hip implants will be used for comparison: the Maxima Cemented Vincula Stem (Víncula, Rio Claro, SP, Brazil) and the Taper Uncemented Primary Stem (Víncula, Rio Claro, SP, Brazil). Patients and evaluators will be blinded to group allocation: the hybrid group (HG) and the uncemented group (UG). A total of 120 patients will be enrolled in this study and randomly allocated to each group in a 1:1 ratio.

### Recruitment and Preoperative Assessment

**Figure 1.**
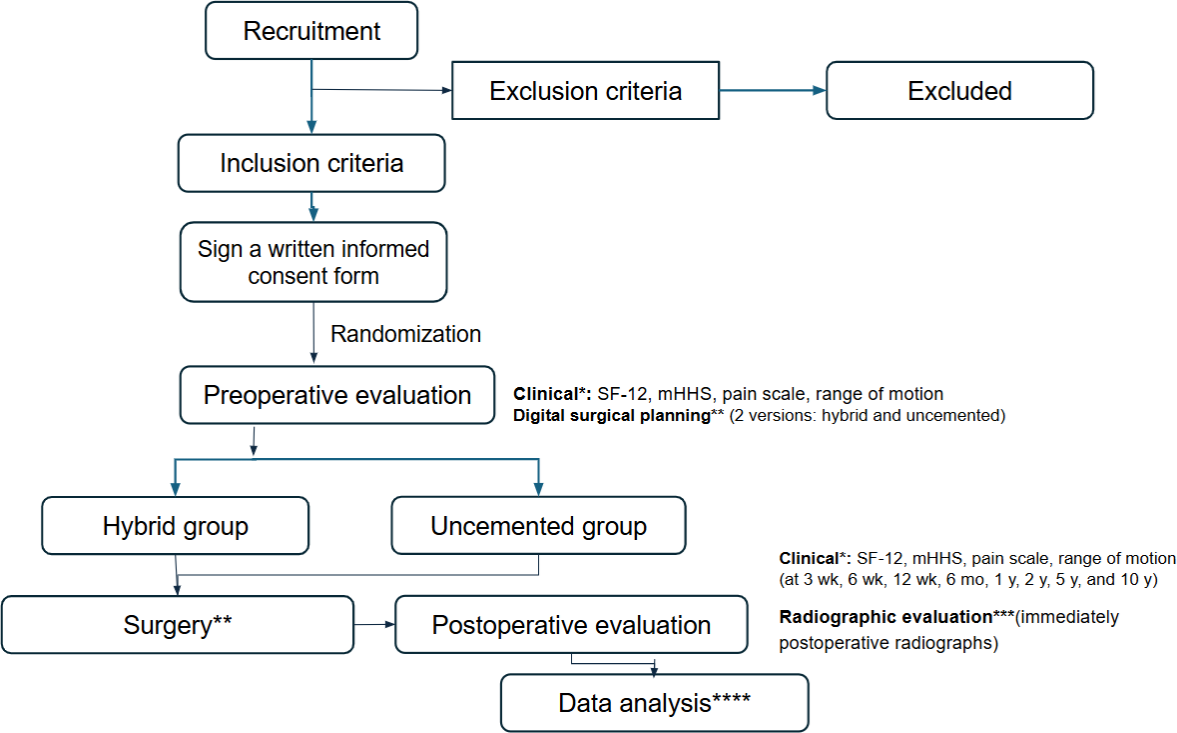
Flowchart of the study protocol. mHHS: modified Harris Hip Score; mo: months; SF-12: 12-Item Short Form Health Survey; wk: weeks; y: years; *: clinical evaluator; **: surgeon; ***: radiographic evaluator; ****: data curator (supervisor).

All the patients ascribed to a waiting list for hip arthroplasty in a hip reconstruction ambulatory of a tertiary teaching hospital in Juiz de Fora-MG, in southeast Brazil (Hospital e Maternidade Therezinha de Jesus) will form the recruitment base for this study. This study was approved by an institutional review board, registered in the Brazilian Registry of Clinical Trials (RBR-263nf22), and assigned the Universal Trial Number U1111-1313-6912. All eligible patients will be informed verbally about the study and its objectives and will be invited to participate (Figure 1). All exclusions will be recorded and reported. Patients who consent to participate will be invited for a consultation when they sign a written informed consent form and will be assigned a sequential study number.

Standard digital anteroposterior pelvis radiographs with calibration spheres will be obtained. A clinical interview and thorough preoperative evaluation will be undertaken by a medical doctor (orthopedic surgery resident). Functional, pain, and QoL questionnaires will be administered at this point, and surgical appointments will be scheduled (Multimedia Appendix 1).

Patient assignment and preoperative evaluations will be conducted in monthly blocks of 12 patients for practical and logistic purposes. Random allocation of patients to each group, the HG and the UG, will be performed at this time using double blinding.

At this stage, the surgeon will be blinded to group allocation and clinical assessment and will perform surgical planning for both uncemented and hybrid procedures using Peekmed software (Peekmed Inc, Braga, Portugal). Peekmed is a highly sensitive tool currently in use that focuses on digital surgical planning to evaluate the accuracy of corrections and to reproduce patient-specific anatomy. Data from both surgical plans will be extracted by the same surgeon using a specially designed digital form (MultimediaAppendices 2  3). Reports of the surgical plans will be printed and kept until surgery; however, only the correct plan, as decided by the random group allocation, will be provided to the surgeon by the assistant researcher before surgery.

### Inclusion and Exclusion Criteria

The inclusion and exclusion criteria are shown in Textbox 1.

Textbox 1. **Inclusion and exclusion criteria.**

**Inclusion criteria**

- Age between 40 and 80 years
- Clinical and radiographic diagnosis of hip arthritis (primary or secondary), unilateral or bilateral
- Both sexes

**Exclusion criteria**

- History of pathological fractures or active, uncontrolled metabolic and/or rheumatic diseases
- Severe hip deformities with extensive acetabular defects, such as severe hip dysplasia
- Previous hip infection

### Withdrawal From the Study

Participants who wish to withdraw after the operation, as instructed in the preparticipation recommendations, may continue with usual treatment at the hospital. In the case of withdrawal at any point after surgery, the data will be considered missing, and data imputation techniques will be evaluated. Data from participants who discontinue the study will remain available in the electronic dataset during revision and will be presented in the results. The study will follow the Consolidated Standards of Reporting Trials (CONSORT) flow chart and tables.

### Randomization

Participants will be randomized and allocated to 2 different groups. On the day the participant signs the written consent form, each participant will receive a unique identification number that will be randomized later. Randomization will be performed in 10 random blocks of 12 participants each using Randomized.org. A computer-generated list will assign participants to either (i) the HG or (ii) the UG. An assistant researcher who is not involved in other study roles will carry out this allocation process. Data related to the randomization list will be kept confidential by the researcher in charge of the randomization. Consequently, both the researcher responsible for outcome evaluations and the participants will be blinded to group allocation.

### Blinding

Before surgery, the surgeon will be blinded to group allocation and will generate radiographic measurements and surgical planning for both uncemented and hybrid surgeries for each patient. All participants will be blinded to the technique utilized. However, a record for medical use will be available, upon request, in the hospital medical file. The surgeon cannot be blinded during the surgical procedure because this is a clinical trial of surgical treatment techniques. To minimize observer bias, all postoperative clinical and functional evaluations will be performed by independent researchers blinded to participant group allocation and postoperative radiographs. Postoperative radiographs will be analyzed by a separate researcher with experience in hip surgery, who will be blinded to preoperative measurements, surgical planning, and postoperative clinical and functional evaluations.

Therefore, blinding was applied in all 3 stages and across all 3 levels, which is known as triple-blinding. The surgeon is blinded to the preoperative evaluation and performs surgical planning without knowledge of which implant will be used. Blinding is broken at the time of surgery. Participants remain blinded throughout the study. Two types of evaluators are involved: an imaging evaluator and a clinical evaluator. The postoperative evaluator, using the X-ray, is blinded to the preoperative evaluators and the postoperative clinical evaluation. The postoperative clinical evaluator is blinded to both group evaluation and radiographic examination. A description of each researcher involved in the study is provided below in Table 1.

**Table 1.** Description of each role in the study.

Research role	Action	How was blinding implemented	Breach of blinding
Surgeon	Independent surgical planning for every patient, generating two archives: hybrid construct and uncemented construct	The surgeon received the digital radiograph of each patient and was unaware of the allocation group	Blinding was relieved only at the time of surgery, when the surgeon received only the applicable copy of the digital plan previously generated, according to the random allocation
Patient	Remained blinded to the allocation group throughout the study	The patient received no information regarding group allocation. The discharge summary from the hospital contained only a reference that he was participating in the THA^a^ study and that details about the implant were available on file upon request	No cases of unblinding were observed
Evaluators			
Clinical evaluators	Applied questionnaires and physical exam, blinded to the allocation group and postoperative radiographs	This group of doctors was called in exclusively for this task during the study	No cases of unblinding were observed
Radiographic evaluators	An orthopedic surgeon analyzed exclusively postoperative radiographs using a custom extraction questionnaire	This evaluator had no access to preoperative radiographs, surgical planning, allocation group, clinical evaluations, or the patient themselves	No cases of unblinding were observed

a THA: total hip arthroplasty.

### Interventions

#### Initial Common Procedure for Both Groups

Patients will be admitted on the day before surgery and will undergo 8 hours of fasting before surgery. Antibiotic prophylaxis (cefazolin 2 g) will be administered 1 hour before anesthetic incision (clindamycin 600 mg will be used as an alternative in cases of allergy). Patients will receive 1 g of tranexamic acid administered intravenously.

Surgery will be performed under neuraxial block (spinal anesthesia) with sedation. Clinical measurement of limb length discrepancy will be estimated by the surgeon with the patient in a supine position and recorded, along with all surgical details, in a specific digital form (Multimedia Appendix 4).

On a regular surgical table, the patient will be positioned in the lateral decubitus, with a hip-positioning device supporting the sacrum and both anterior superior iliac spines. Limb length will be reassessed at this position.

Routine aseptic cleansing and preparation will be performed, and sterile surgical fields will be positioned. The limb to be operated on will be degermed with polyvinylpyrrolidone iodine solution (chlorhexidine will be used instead for patients with allergy), and excess solution will be removed with alcohol. Antisepsis with alcoholic polyvinylpyrrolidone iodine solution will be performed by the surgeon, and sterile drapes will be positioned.

The same surgeon will conduct the surgery on every patient using a standard posterolateral approach. The piriformis tendon, along with other external rotators, will be divided from the femur and tagged with an Ethibond® 5 sutures for later transosseous reinsertion. The capsule will be opened with an L-shaped incision and tagged with an Ethibond® 5 running suture for later reinsertion. Posterior hip dislocation and femoral neck osteotomy, according to the surgical plan, will then be executed with a power saw.

Preparation of the acetabulum will begin with full exposure using a long Homann retractor placed anteriorly and inferiorly. A 4.0 Steinman pin will be placed superiorly for soft tissue retraction. Another 3.5 Steinman pin will be placed between the hip capsule and the posteroinferior labrum in the ischium to complete exposure.

The acetabular labrum, periacetabular osteophytes, round ligament and pulvinar tissue, and curtain osteophytes exposing the fovea will be removed. A successive reaming of the acetabulum will be done with a high-torque drill to the planned diameter and adequate bone contact. The prosthesis will be tested to verify positioning and adequate press fit (with a target inclination of 45° and anteversion of 20°). Subsequently, an uncemented acetabular component (Phenon poly II, Vincula, Rio Claro, Brazil) will be inserted through impaction using the press-fit technique. Additional fixation with 2 to 3 acetabular screws will be used as needed by the surgeon, depending on the perception of the press-fit and bone quality. Insertion of a highly cross-linked polyethylene liner will be performed through impaction, followed by confirmation of system stability.

After performing the procedure on the acetabulum, patients will undergo the femoral procedure according to the group to which they were allocated.

#### Femoral Procedure in the HG

Patients allocated for hybrid arthroplasty will undergo a surgical procedure consisting of positioning the femur by internal rotation of 90° and flexion of the hip. The Schumacher-type retractors will be positioned under the anterior surface of the femoral neck, and the remaining capsule and tendons of the piriform fossa will be resected. Following this, the metaphyseal bone with a box-type chisel will be removed, and a cylindrical femoral starting broach will be used to enlarge the canal. According to the surgical plan, the femoral canal will be prepared with successive femoral rasps until adequate stability and restoration of the anatomical parameters of offset and length are achieved.

Following the surgical plan, a femoral neck and head test will be done. Hip reduction, stability test at the extremes of the range of motion (ROM), assessment of limb length, soft tissue tension, and dislocation of the test prosthesis will be done, and the hip reduced to assess stability, limb length restoration, and soft tissue tension. After ensuring adequate size, the surgeon will dislocate the hip joint again and mark it with electrocautery for guidance during definitive implant positioning. Further removal of bone at the Gruen zone I will be undertaken with a curette for adequate cement penetration. A cement restrictor will then be placed, obliterating the femoral canal 2 cm distal to the tip of the femoral stem. Copious irrigation of the canal and thorough cleansing and drying will be performed to remove all blood and medullary content. Cementation of the femoral canal using a retrograde technique with a cement pistol and two cements will be done under pressure. The polished double wedge cemented stem (Máxima, Vincula, Rio Claro, Brazil) will be introduced at a constant speed with a distal centralizer to the planned position. The target anteversion will be 15° with central alignment to the femoral axis.

#### Femoral Procedure in the UG

Patients allocated to uncemented arthroplasty will undergo a surgical procedure consisting of positioning the femur in 90° of internal rotation with hip flexion. Schumacher-type retractors will be placed beneath the anterior surface of the femoral neck, and the remaining capsule and tendons of the piriform fossa will be resected. Subsequently, the metaphyseal bone will be removed using a box-type chisel, and a cylindrical femoral starting broach will be used to enlarge the canal. The femoral canal will be reamed with successive femoral rasps, according to the plan, until adequate stability and restoration of the anatomical parameters of offset and length.

A testing femoral head will be assembled, and the hip will be reduced. Hip stability, limb length restoration, and soft tissue tension will be assessed. After ensuring adequate size, the surgeon will dislocate the hip joint again and mark it with electrocautery for guidance during definitive implant positioning. The uncemented titanium triple wedge plasma-sprayed femoral stem (Taper, Vincula, Rio Claro, Brazil) will be introduced by hammering with a press-fit technique down to the planned position. The target anteversion will be 15° with central alignment to the femoral axis.

#### Final Common Procedure for Both Groups

A metallic femoral head will be introduced and impacted on the Morse taper of the stem. The hip will then be reduced, followed by a final check of stability at the extremes of the ROM, as well as evaluation of limb length and soft tissue tension.

For closure, 2 holes will be drilled in the greater trochanter using a 2.5-mm drill for reinsertion of the external rotators and posterior hip capsule. Ethibond sutures will be tightened for a transosseous repair of the external rotator capsule in anatomical position. The fascia lata will be closed with X-shaped Vicryl® 2.0 separate stitches. The subcutaneous layer will be sutured with inverted Vicryl® 2.0 separate stitches. Finally, a suture of the skin with intradermal stitches (continuous suture) of Nylon 2.0 and a sterile dressing with gauze and adhesive tape will be done.

### Postoperative Care

In both groups, early active motion of the operated limb will be permitted as soon as motor function recovers from anesthesia. All participants will be instructed to begin immediate knee flexion and extension exercises, ankle and foot movements, and active calf pump exercises. No orthotic devices or physical positional measures will be utilized to restrict hip motion. However, all patients will be advised to avoid flexion, adduction, and internal rotation of the operated hip, or sitting in low chairs for at least 3 months. Walking with 2 crutches with weight-bearing as tolerated will be stimulated on the first postoperative day until the follow-up visit at 21 days, and patients will be discharged for ambulatory care as soon as ambulation is possible with comfort. Sports and activities on extreme hip motions will be discouraged for at least 3 months.

### Primary Outcomes

The primary outcomes of this study will be patient-reported function and complications.

Clinical evaluation will comprise the measurement of hip ROM to assess joint mobility, and the HHS questionnaire will be applied to assess function. Hip ROM will be measured using the goniometer, with the patient lying supine, to determine joint mobility. Hip function will be assessed using the HHS, which is a THA-specific questionnaire composed of patient-reported function and physician-assessed joint ROM, including pain, function, deformity, and mobility assessment [19,20]. The HHS is scored up to 100 points, where each section has a different score, and after applying the questionnaire, all scores are summed up. Scores below 70 are considered low, scores between 70 and 80 are considered fair, scores between 80 and 90 are considered good, and scores between 90 and 100 are considered excellent [19].

The incidence of complications (during and after surgery) will be extracted from an intraoperative questionnaire completed by the surgeon, an immediate postoperative questionnaire reported by the patient, and a special severe adverse effect questionnaire, which will be filled whenever the patient requires readmission or additional surgery.

### Secondary Outcomes

Secondary outcomes will include comparisons of QoL, joint mobility, function, satisfaction, and pain before and after the surgery, as well as radiographic parameters extracted from the immediate postoperative radiograph. The annual frequency of revision surgeries will be recorded.

QoL will be evaluated using 12-Item Short Form Health Survey version 2 questionnaire [21]. QoL is defined as an individual’s perception of their position in life in the context of the culture and value systems in which they live and about their goals, expectations, standards, and concerns [21]. The 12-Item Short Form Health Survey is an instrument for measuring QoL composed of 12 items that assess eight different dimensions of influence on QoL, considering the individual’s perception of aspects of their health in the last 4 weeks [21]. Each dimension’s score is summed up, and the final score can be between 0 and 100; the higher the score, the better the QoL [22].

Participant satisfaction assessment will be measured using a 10-point numerical rating scale ranging from 1 (very dissatisfied) to 10 (completely satisfied) [23].

Other aspects of health, such as pain, will be evaluated through the Visual Analog Scale. The Visual Analog Scale provides a simple and efficient measure of pain intensity and is used when a fast pain index that can be assigned to a numerical value is needed [24].

Radiographic parameters will include the ability to restore anatomical normality parameters on postoperative radiographs according to the surgical plan (namely the limb length discrepancy, the horizontal off-set of the hip, difference between the back of the prosthesis and the tip of the trochanter, inclination of the acetabular component, and the difference between offset obtained to the normal side) all of this using the Peekmed surgical planning software.

The annual frequency of revision surgery will be observed starting from 1 year after surgery and annually until 5 years, and up to 10 years.

All questionnaires will be administered by examiners blinded to the surgical procedure. Questionnaires will be completed using digital devices (computer or tablet).

### Data Collection and Access

Participant data will be collected using study forms (MultimediaAppendices 1^4^) and stored on a designated Google Drive, which will be used as the study repository. A copy of all the files will be stored on an external hard drive for backup. The research supervisor will audit completion of the electronic spreadsheet and will be responsible for data safety and accuracy. Incorrect or missing data will be assessed by this researcher, who will not be involved in other tasks in the study, and corrected when necessary. Data from participants who discontinue the study will remain available in Google Drive for the reviewers and will be presented in the results, CONSORT flowchart, and tables. Study data will be stored for at least 5 years after completion of the study.

### Level of Pragmatism

**Figure 2.**
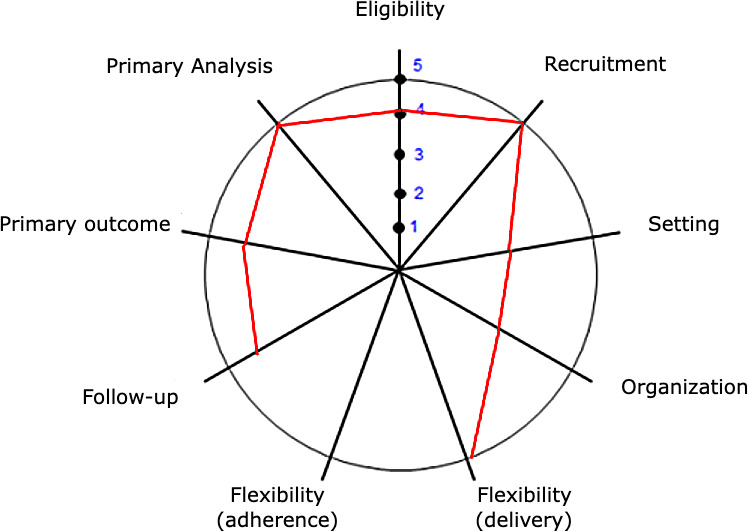
ePragmatism level wheel. 1: very explanatory; 2: rather explanatory; 3: equally explanatory/pragmatic; 4: rather pragmatic; 5: very pragmatic.

The level of pragmatism is shown in Figure 2 and detailed in Table 2.

**Table 2.** Domains and score of pragmatism level.^a^

Domains	Score	Rationale
1. Eligibility—Who is selected to participate in the trial?	4	All patients already had an indication for total hip arthroplasty in the outpatient clinic. Exclusions were limited to cases in which the disease could compromise the results because of acetabular outcome (such as severe hip dysplasia) or when there was a known indication for other specific implants (eg, ceramic femoral heads for patients younger than 40 years old).
2. Recruitment—How are participants recruited into the trial?	5	Patients were selected from the public waiting list and referred by the health department and/or spontaneous demand. The study was announced, but the waiting list at the beginning of the study already included the entire projected population.
3. Setting—Where is the trial being done?	3	Despite being a tertiary teaching hospital, currently, this is the most common setting to offer this treatment in the public system (Sistema Único de Saúde) in Brazil
4. Organization—What expertise and resources are needed to deliver the intervention?	3	Because of the study, a dedicated set of instruments was kept in the hospital for consistency, which does not usually occur in the routine care. However, the surgical team, operating rooms, and available equipment are the same as the usual care.
5. Flexibility (delivery)—How should the intervention be delivered?	5	Surgery will be undertaken as planned, in the same manner as the usual technique used at the hospital over the last 10 years
6. Flexibility (adherence)—What measures are in place to make sure participants adhere to the intervention?	—	Surgical intervention (to be left blank)
7. Follow-up—How closely are participants followed up?	4	Patient visits to the clinic will follow the same pattern as the usual care. Because diverse questionnaires need to be applied, each visit is expected to be longer than routine consultations
8. Outcome—How relevant is it to participants?	4	Pain, satisfaction, quality of life, function, and need for revision are highly relevant to patients. However, radiographic parameters will also be measured, which are mostly important only for surgeons
9 Analysis—To what extent are all data included?	5	After surgery, all data will be collected and analyzed. An intention-to-treat approach is planned if necessary

a 1: very explanatory; 2: rather explanatory; 3: equally explanatory / pragmatic; 4: rather pragmatic; 5: very pragmatic.

### Sample Size

The sample size was calculated using the Statulator Sample Size Calculation tool [25]. To disprove the null hypothesis of equality, assuming a pooled SD of 10.5 units for the HHS [6], the study would require a sample size of 60 participants per each group (ie, a total sample size of 120 for equal group sizes) to achieve a power of 80% and a significance level of .05 (2-sided) for detecting a true difference in means of 5.39 units between the test and the reference groups (ie, 56.9 vs 62.3).

### Statistical Analysis

Results will be presented descriptively, with continuous data expressed as means (SD) and medians, and categorical data will be presented as means or percentages. For inferential analysis, the normality of the variables will be assessed using the Kolmogorov-Smirnov test. Dichotomous variables will be analyzed using the Fisher exact test and chi-square test, and continuous variables will be analyzed using the *t* test. Demographic data will be analyzed using ANOVA test, and the chi-square test will be used for the categorical data. In cases of non-normal distribution, appropriate nonparametric tests will be used according to the nature of the variable.

Primary outcomes, complications, and reoperations will be analyzed at 48 weeks. Paired and subgroup analyses are foreseen between periods of 3, 6, 24, and 48 weeks for the described outcomes. Results will be reported with 95% CI.

All statistical tests will be 2-sided, with a significance level of *P*=.05. Data will be analyzed using the Online Statistics Calculator, DATAtab/Numiqo (2025) [26]. Data from participants who receive additional treatments (eg, revision surgery for fractures) during study participation will be retained for intention-to-treat analysis.

### Ethical Considerations

This study was approved by the Institutional Review Board of the Faculty of Medical and Health Sciences of Juiz de Fora–Suprema (CAAE: 82413124.4.0000.5103). Any important protocol modifications will be communicated to the ethics committee. Study results will be communicated through the paper publication and will be explained to participants who wish to know their individual results. No compensation will be provided to any participant. All digital records will be stored on a private, password-protected Google Drive. Access to each specific folder will be granted only to the researchers to the extent of their participation. Only the study supervisor will be granted full access to the whole database during the study. Participants must sign a written informed consent form before they are invited for a consultation.

## Results

The study started in October 2024. As of April 2025, 72 participants have been enrolled and operated on, corresponding to 60% of the target population. As the study timeline is being maintained, the first data analysis of the complete population was performed in November 2025, reporting population characteristics, radiographic and intraoperative findings, and early complications. The first results are expected to be published in the first half of 2026.

## Discussion

### Background and Rationale

In Brazil, up to 75% of the population relies exclusively on the public health system (Sistema Único de Saúde [SUS]). From 2008 to 2015, 166,365 total hip replacements were performed only in SUS [27]. After 2012, the number of uncemented and hybrid constructs surpassed the cemented replacements in Brazil [19].

Designed as a universal, integral, and equitable system, SUS should consider the cost-effectiveness of the treatments provided [27]. While total hip replacement has been proven cost-effective in many countries around the world [28], such studies were carried out using data from implants that are either unavailable in Brazil or are much more expensive due to importation fees and taxes. Therefore, although modern national implants have become available and good clinical outcomes have been reported in some studies [15-17]here are no level 1 studies currently available, to date, comparing such implants. Additionally, this study introduces an innovation by being the first to use Peekmed, an artificial intelligence–based preoperative planning software for orthopedic surgery.

Besides clinical information as to whether the Brazilian population should have better outcomes using hybrid or uncemented total hip replacement, the conclusion of this study may also be instrumental for future economic studies applicable to SUS and the private health sector in Brazil.

This study is expected to have several limitations: the fact that it was conducted in one hospital and by one surgeon may raise questions about external validity and reproducibility in other scenarios. On the other hand, this feature provides better homogeneity, increases internal validity, and consistency of results. To minimize this external validity limitation, we used a pragmatic approach to mitigate the potential bias of a very controlled setting. Another potential conflict is that the company that makes the implants provided them at a discount rate and gave equipment to the study team. Even though it is called an “unrestricted agreement,” this still creates a risk of potential conflict of interest because the results could benefit that company. The researchers received no personal benefits, salaries, or incentives, which is relevant for this subject. Additionally, each extract of information was collected independently by the researchers, and the complete database was only accessible to one independent data curator (the research supervisor), who was only responsible for that task and had no role in data collection. An additional measure to reduce bias was the use of implants from the same manufacturer, thereby minimizing the risk of conflict of interest in the comparison between implants. Nevertheless, a potential influence on the overall results cannot be entirely excluded, as a purchase discount was applied. The blinding of the surgeon, the evaluators, and the patients in different stages of the study also reinforces the commitment to diminish biases and increase the independence of the results.

### Conclusion

This study is a prospective, randomized, pragmatic clinical trial that uses blinding to evaluate national implants for both hybrid arthroplasty and uncemented arthroplasty. It is the first study with adequate power to prospectively evaluate the outcomes of each surgical procedure in Brazil. Successful completion of this study may provide clinical evidence on the performance of national implants, as well as showing the preferred implant construct (hybrid or uncemented), if any, for hip replacement in Brazil.

## Supplementary material

10.2196/79721 Multimedia Appendix 1 Functional, pain, and quality of life questionnaires.

10.2196/79721 Multimedia Appendix 2 Surgical plan 1.

10.2196/79721 Multimedia Appendix 3 Surgical plan 2.

10.2196/79721 Multimedia Appendix 4 Clinical measurement of limb length discrepancy.

10.2196/79721 Checklist 1 SPIRIT checklist.

## Abbreviations

CONSORT: Consolidated Standards of Reporting Trials
HG: hybrid group
HHS: Harris Hip Score
QoL: quality of life
RCT: randomized controlled trial
ROM: range of motion
SPIRIT: Standard Protocol Items: Recommendations for Interventional Trials
SUS: Sistema Único de Saúde
THA: total hip arthroplasty
UG: uncemented group
