# Supplementary material for: Comparison of Uncemented and Hybrid Hip Arthroplasty: Protocol for a Brazilian Randomized Controlled Trial
Source: JMIR Res Protoc. 2026 Mar 16;15:e79721. doi: 10.2196/79721 (PMC12991186; doi:10.2196/79721)
Supplement: Multimedia Appendix 1 [file resprot-v15-e79721-s001.PDF]

EBRA-HIP120 - PRÉ OPERATÓRIO

* Indica uma pergunta obrigatória

1. 1. Número do paciente no estudo *

2. 2. Nome completo do paciente: *

3. 3. Número do prontuário HMTJ *

4. 4. Data de Nascimento *

   Exemplo: 7 de janeiro de 2019

5. 5. Idade *

6. 6. Sexo *

   Marcar apenas uma oval.

   ◯ Masulino
   ◯ Feminino

7. 7. Peso (em kilogramas = XX,x Kg) *

____

8. 8. Altura (em metros= X,xx m) *

____

9. 9. Tempo de evolução dos sintomas (em anos ou meses = especificar) *

____

10. 10. Tempo aguardando cirurgia (em anos ou meses = especificar) *

____

11. 11. Qual quadril dói: *

Marcar apenas uma oval.

- ( ) APENAS O DIREITO
- ( ) APENAS O ESQUERDO
- ( ) BILATERAL, O DIREITO MAIS QUE O ESQUERDO
- ( ) BILATERAL, O ESQUERDO MAIS QUE O DIREITO
- ( ) BILATERAL, AMBOS DOEM IGUAL

EXAME CLÍNICO (Para DÉFICIT ANOTAR COM (-))

12. 12- MOBILIDADE DO QUADRIL= FLEXÃO (EM GRAUS) *

____

13. 13- MOBILIDADE DO QUADRIL= EXTENSÃO (MEDIR NA POSIÇÃO DE THOMAS) (EM GRAUS) *

____

14. 14- MOBILIDADE DO QUADRIL= ROTAÇÃO INTERNA (MEDIR NA POSIÇÃO DE FLEXÃO MÁXIMA) (EM GRAUS) *

____

15. 15- MOBILIDADE DO QUADRIL= ROTAÇÃO EXTERNA (MEDIR NA POSIÇÃO DE FLEXÃO MÁXIMA) (EM GRAUS) *

____

16. 17- MOBILIDADE DO QUADRIL= ABDUÇÃO (MEDIR NA POSIÇÃO DE EXTENSÃO) (EM GRAUS) *

____

17. 18- MOBILIDADE DO QUADRIL= ADUÇÃO (MEDIR NA POSIÇÃO DE EXTENSÃO) (EM GRAUS) *

____

Seção sem título

ESCALA VISUAL ANALÓGICA DA DOR

18. QUAL A INTESIDADE DA SUA DOR NO QUADRIL? (DE 0 (SEM DOR) A 10 (MAIOR DOR QUE VOCÊ JÁ SENTIU). *

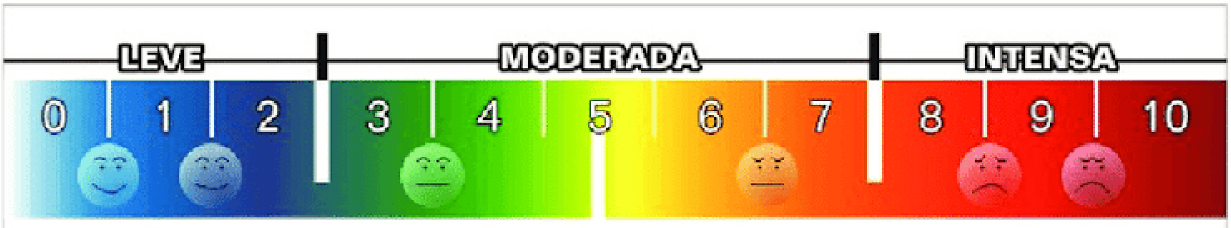

Marcar apenas uma oval.

	1	2	3	4	5	6	7	8	9	10	
LEVI	◯	◯	◯	◯	◯	◯	◯	◯	◯	◯	INTENSA

QUESTIONÁRIO SF-12

Instruções: Queremos saber sua opinião sobre sua saúde. Essa informação nos ajudará a saber como o(a) sr(a) se sente e como é capaz de fazer suas atividades do dia a dia. Responda cada questão indicando a resposta certa. Se está em dúvida sobre como responder a questão, por favor, responda da melhor maneira possível. Marque apenas uma opção em cada pergunta.

19. 1. Em geral, o(a) sr(a) diria que sua saúde é: *

Marcar apenas uma oval.

- Excelente (1 ponto)
- Muito boa (2 pontos)
- Boa (3 pontos)
- Regular (4 pontos)
- Ruim (5 pontos)

AS PERGUNTAS SEGUINTES SÃO SOBRE COISAS QUE O(A) SR(A) FAZ NA MÉDIA, NO SEU DIA A DIA (DIA TÍPICO/COMUM)

O(a) sr(a) acha que sua saúde, agora, o dificulta de fazer algumas coisas do dia a dia, como por exemplo:

20. 2. Atividades médias (como mover uma cadeira, fazer compras, limpar a casa, trocar de roupa)? *

Marcar apenas uma oval.

- Sim, dificulta muito (1 ponto)
- Sim, dificulta pouco (2 pontos)
- Não, não dificulta de modo algum (3 pontos)

21. 3. O(a) sr(a) acha que sua saúde, agora, o dificulta de fazer algumas coisas do dia a dia, como por exemplo: subir três ou mais degraus de escada?

Marcar apenas uma oval.

- Sim, dificulta muito (1 ponto)
- Sim, dificulta pouco (2 pontos)
- Não, não dificulta de modo algum (3 pontos)

Sem título

Durante as últimas 4 semanas, o(a) sr(a) teve algum dos seguintes problemas com seu trabalho ou em suas atividades do dia a dia, como por exemplo:

22. 4. Fez menos do que gostaria, por causa de sua saúde física? *

Marcar apenas uma oval.

- Sim (1 ponto)
- Não (2 pontos)

23. 5. Durante as últimas 4 semanas, o(a) sr(a) teve algum dos seguintes problemas com seu trabalho ou em suas atividades do dia a dia, como por exemplo: sentiu-se com dificuldade no trabalho ou em outras atividades, por causa de sua saúde física? *

Marcar apenas uma oval.

- Sim (1 ponto)
- Não (2 pontos)

Sem título

Durante as últimas 4 semanas, o(a) sr(a) teve algum dos seguintes problemas, como por exemplo:

24. 6. Fez menos do que gostaria, por causa de problemas emocionais? *

Marcar apenas uma oval.

- Sim (1 ponto)
- Não (2 pontos)

25. 7. Durante as últimas 4 semanas, o(a) sr(a) teve algum dos seguintes problemas, como por exemplo: deixou de fazer seu trabalho ou outras atividades cuidadosamente como de costume, por causa de problemas emocionais?

Marcar apenas uma oval.

- Sim (1 ponto)
- Não (2 pontos)

26. 8. Durante as últimas 4 semanas, alguma dor atrapalhou seu trabalho normal (tanto o trabalho de casa como o de fora de casa)?

Marcar apenas uma oval.

- Não, nem um pouco (1 ponto)
- Um pouco (2 pontos)

ESTAS QUESTÕES SÃO SOBRE COMO O(A) SR(A) SE SENTE E COMO AS COISAS TÊM ANDADO PARA O (A) SR(A), DURANTE AS 4 ÚLTIMAS SEMANAS.

Quanto tempo durante as últimas 4 semanas:

27. 9. O(a) Sr(a) tem se sentido calmo e tranquilo

Marcar apenas uma oval.

- Todo o tempo (1 ponto)
- A maior parte do tempo (2 pontos)
- Uma boa parte do tempo (3 pontos)
- Alguma parte do tempo (4 pontos)
- Uma pequena parte do tempo (5 pontos)
- Nem um pouco do tempo (6 pontos)

28. 10. Quanto tempo durante as últimas 4 semanas: o(a) sr(a) teve bastante energia?

Marcar apenas uma oval.

- Todo o tempo (1 ponto)
- A maior parte do tempo (2 pontos)
- Uma boa parte do tempo (3 pontos)
- Alguma parte do tempo (4 pontos)
- Uma pequena parte do tempo (5 pontos)
- Nem um pouco do tempo (6 pontos)

29. 11. Quanto tempo durante as últimas 4 semanas: o(a) sr(a) sentiu-se desanimado e deprimido?

Marcar apenas uma oval.

- Todo o tempo (1 ponto)
- A maior parte do tempo (2 pontos)
- Uma boa parte do tempo (3 pontos)
- Alguma parte do tempo (4 pontos)
- Uma pequena parte do tempo (5 pontos)
- Nem um pouco do tempo (6 pontos)

30. 12. Durante as últimas 4 semanas, em quanto do seu tempo a sua saúde ou problemas emocionais atrapalharam suas atividades sociais, tais como: visitar amigos, parentes, sair, etc?

Marcar apenas uma oval.

- Todo o tempo (1 ponto)
- A maior parte do tempo (2 pontos)
- Uma boa parte do tempo (3 pontos)
- Alguma parte do tempo (4 pontos)
- Uma pequena parte do tempo (5 pontos)
- Nem um pouco do tempo (6 pontos)

Instrumento de avaliação do Quadril de Harris (**HARRIS HIP SCORE)**

SOBRE A FUNÇÃO E SINTOMAS DO SEU QUADRIL:

31. 1- Dor *

Marcar apenas uma oval.

- A) Nenhuma ou ignora (44)
- B) Leve, ocasional, não compromete as atividades (40)
- C) Dor fraca, não interfere nas atividades médias, raramente dor moderada com atividade pouco comum, pode tomar aspirina (30)
- D) Dor moderada, tolerável, mas faz concessões à dor. Alguma limitação nas atividades comuns ou no trabalho. Ocasionalmente precisa de analgésico mais forte que a aspirina (20)
- E) Dor acentuada, atividades bastante limitadas (10)
- F) Invalidez total, deficiente, dor na cama, não sai da cama (0)

II- Função

A. Marcha

32. 1. Claudicação: *

Marcar apenas uma oval.

- a) Nenhuma (11)
- b) Leve (8)
- c) Moderada (5)
- d) Forte (0)

33. 2. Apoio: *

Marcar apenas uma oval.

- a) Nenhum (11)
- b) Bengala para caminhadas longas (7)
- c) Bengala a maior parte do tempo (5)
- d) Uma muleta (3)
- e) Duas bengalas (2)
- f) Duas muletas (0)
- g) Não consegue andar (especificar o motivo) (0)

34. Se selecionado "g) Não consegue andar (especificar o motivo) (0)", favor especificar o motivo:

____

____

____

____

____

35. 3. Distância que consegue andar: *

Marcar apenas uma oval.

- a) limitada (11)
- b) 6 quarteirões (8)
- c) 2-3 quarteirões (5)
- d) Apenas dentro de casa (2)
- e) Da cama até a cadeira (0)

II- Função

B. Atividades

36. 1. Subir e descer escadas: *

Marcar apenas uma oval.

- a. Normalmente, sem usar um corrimão (4)
- b. Normalmente, usando um corrimão (2)
- c. De qualquer forma (1)
- d. Não consegue usar escadas (0)

37. 2. Calçar sapato e meia: *

Marcar apenas uma oval.

- a) Com facilidade (4)
- b) Com dificuldade (2)
- c) Não consegue (0)

38. 3. Sentar: *

Marcar apenas uma oval.

- ⬭ a) Senta-se confortavelmente em cadeira comum durante uma hora (5)
- ⬭ b) Senta-se em cadeira alta durante meia hora (3)
- ⬭ c) Não consegue sentar-se de forma confortável em nenhuma cadeira (0)

39. 4- Tomar transporte público *

Marcar apenas uma oval.

- ⬭ Sim (1)
- ⬭ Não

40. III. Considera-se não haver pontos de deformidade quando o paciente apresenta * (Marque todos que estiverem presentes).

Marcar apenas uma oval.

- ⬭ A) Contratura em flexão fixa inferior a 30°
- ⬭ B) Contratura em adução fixa inferior a 10°
- ⬭ C) Contratura em rotação interna fixa em extensão inferior a 10°
- ⬭ D) Discrepância no comprimento dos membros inferior a 3,2 centímetros

IV. Amplitude de movimento (o valor do índice é calculado pela multiplicação dos graus de movimento possíveis de cada arco pelo respectivo índice)

41. A. Flexão *

Marcar apenas uma oval.

- ⬭ 0 a 45 graus (1,0)
- ⬭ 45 a 90° (0,6)
- ⬭ 90 a 110° (0,3)

42. B. Abdução *

Marcar apenas uma oval.

- 0 a 15° (0,8)
- 15 a 20° (0,3)

43. C. Rotação externa na extensão *

Marcar apenas uma oval.

- 0 a 15° (0,4)
- Mais de 15° (0)

44. D. Rotação interna na extensão *

Marcar apenas uma oval.

- Qualquer (0)

45. E. Adução *

Marcar apenas uma oval.

- 0 a15° (0,2)

Este conteúdo não foi criado nem aprovado pelo Google.

Google Formulários
