# Supplementary material for: Comparison of Uncemented and Hybrid Hip Arthroplasty: Protocol for a Brazilian Randomized Controlled Trial
Source: JMIR Res Protoc. 2026 Mar 16;15:e79721. doi: 10.2196/79721 (PMC12991186; doi:10.2196/79721)
Supplement: Multimedia Appendix 3 [file resprot-v15-e79721-s003.PDF]

Pré Planejamento ATQ NÃO CIMENTADA

* Indica uma pergunta obrigatória

1. Número do paciente operado *

____

2. MAGNIFICAÇÃO DO PLANEJAMENTO (notação XX,xx%) *

____

3. Lado acometido

Marcar apenas uma oval.

( ) Direito
( ) Esquerdo
( ) Bilateral

4. Lado operado nesta cirurgia *

Marcar apenas uma oval.

( ) Direito
( ) Esquerdo

5. Provável etiologia *

Marcar apenas uma oval.

- Osteonecrose
- Displasia
- Sequela de epifisiólise
- Sequela de Perthes
- Coxartrose primária
- Não é possível definir

6. Morfologia acetabular

Marcar apenas uma oval.

- NORMAL
- DISPLASICO
- COXA PROFUNDA
- OTOPELVE
- OSTEOFITOSE MARGINAL
- OSTEOFITO EM CORTINA COM LATERALIZAÇÃO DA CABEÇA
- CISTOS GRANDES
- DEFEITO ÓSSEO
- OUTROS

7. Necessidade de enxerto ósseo NO ACETÁBULO?

Marcar apenas uma oval.

- Sim
- Não

8. Fratura prévia?

Marcar apenas uma oval.

- ⬭ Sim, COM SÍNTESE
- ⬭ Sim, SEM SÍNTESE
- ⬭ Não

9. Comprimento MMIIs

Marque todas que se aplicam.

- ☐ D > E
- ☐ E > D
- ☐ D = E

10. Discrepância entre os comprimentos dos membros (linha bi-isquiática ao trocanter menor em milímetros)/ (usar (+) para membro acometido alongado e (-) para membro acometido encurtado):

____

11. Offset do lado não acometido (em milímetros) *

____

12. Espessura do canal medular (milímetros):

____

13. Classificação de Dorr:

Marcar apenas uma oval.

- ⬭ Tipo A (cortical espessa + canal medular estreito)
- ⬭ Tipo B (perda óssea cortical + alargamento do canal intramedular)
- ⬭ Tipo C (cortical fina + canal medular largo)

Pós Planejamento ATQ NÃO CIMENTADA

14. Offset horizontal (milímetros):

____

15. Distância horizontal da gota de lágrima ao centro de rotação (milímetros):

____

16. Distância vertical da linha da gota de lágrima ao centro de rotação (milímetros):

____

17. Distância da ponta do trocanter ao dorso da prótese (milímetros):

____

18. Tamanho do acetábulo (milímetros):

____

19. Inclinação do componente acetabular (em graus):

____

20. Tamanho do fêmur (não-cimentado):

Marcar apenas uma oval.

- 6
- 7
- 8
- 9
- 10
- 11
- 12
- 13
- 14
- 16
- 18

21. Tamanho da cabeça (milímetros):

____

22. Tamanho do colo:

Marcar apenas uma oval.

- Curto
- Médio
- Longo
- X-longo

23. MEDIDA DA DISCREPÂNCIA AO FIM DO PLANEJAMENTO (em milímetros) / (usar (+) para membro acometido alongado e (-) para membro acometido encurtado): *

____

Este conteúdo não foi criado nem aprovado pelo Google.

Google Formulários
