# Supplementary material for: Comparison of Uncemented and Hybrid Hip Arthroplasty: Protocol for a Brazilian Randomized Controlled Trial
Source: JMIR Res Protoc. 2026 Mar 16;15:e79721. doi: 10.2196/79721 (PMC12991186; doi:10.2196/79721)
Supplement: Multimedia Appendix 4 [file resprot-v15-e79721-s004.PDF]

EBRA-HIP120 - INTRAOPERATÓRIO

* Indica uma pergunta obrigatória

1. 1. Número do paciente operado *

   ____

2. 2. Discrepância clínica estimada anestesiado (em milimetros) *

   ____

3. 3. Qual foi o tempo cirúrgico? (minutos) *

   ____

4. 4. Quais foram as dificuldades técnicas durante o procedimento?

   Marque todas que se aplicam.

   - [ ] OBESIDADE
   - [ ] CONTRATURA DE PARTES MOLES
   - [ ] OSTEOPOROSE GRAVE
   - [ ] ESCLEROSE OSSEA
   - [ ] CANAL ESTREITO
   - [ ] NECESSIDADE DE OSTEOTOMIA DO COLO INSITU
   - [ ] INSTABILIDADE APÓS REDUÇÃO DO TESTE
   - [ ] MUITOS OSTEOFITOS
   - [ ] DIFICULADE DE REDUÇÃO
   - [ ] NÃO HOUVE

5. 5. Quais foram as complicações durante o procedimento?

Marcar apenas uma oval.

- FRATURA DO ACETABULO
- PRESS-FIT ACETABULAR INADEQUADO
- FRATURA DO FEMUR INCOMPLETA (SEM OSTEOSSINTESE)
- FRATURA DO FEMUR COM NECESSIDADE DE SÍNTESE
- SANGRAMENTO EXCESSIVO
- COMPLICAÇÃO CLINICA
- COMPLICAÇÃO ANESTESICA
- NÃO HOUVE

6. 6. DIFERENÇA EM MILIMETROS DO ACETABULO PLANEJADO DO IMPLANTADO? *

____

7. 7. HOUVE DIFERENÇA ENTRE O FÊMUR PLANEJADO DO IMPLANTADO?

Marcar apenas uma oval.

- Sim
- Não

8. 8- FÊMUR CIMENTADO (QUAL A DIFERENÇA DO PLANEJADO DO IMPLANTADO)

Marcar apenas uma oval.

- IMPLANTADO COM MESMO OFFSET, DIFERERENTE 1 TAMANHO
- IMPLANTADO COM MESMO OFFSET, DIFERENTE 2 TAMANHOS
- IMPLANTADO UM OFSET MAIOR
- IMPLANTADO UM OFSET MENOR
- MESMO TAMANHO
- NÃO SE APLICA

9. 9. HOUVE MUDANÇA NO OFF-SET NÃO CIMENTADO PLANEJADO *

Marcar apenas uma oval.

- SIM
- Não
- NÃO SE APLICA

10. 10- FÊMUR NÃO-CIMENTADO (QUAL A DIFERENÇA DO PLANEJADO DO IMPLANTADO)

Marcar apenas uma oval.

- IMPLANTADO UM NÚMERO MAIOR
- IMPLANTADO DOIS NÚMEROS MAIOR
- IMPLANTADO TRES NÚMEROS MAIOR
- IMPLANTADO QUATRO NÚMEROS MAIOR
- IMPLANTADO UM NÚMERO MENOR
- IMPLANTADO DOIS NÚMEROS MENOR
- IMPLANTADO TRES NÚMEROS MENOR
- IMPLANTADO QUATRO NÚMEROS MENOR
- MESMO TAMANHO
- NÃO SE APLICA

11. 11. Discrepância clínica estimada anestesiado (em milimetros) *

____

12. 12- OBSERVAÇÕES DO CIRURIÃO

____
____
____
____
____

Este conteúdo não foi criado nem aprovado pelo Google.

Google Formulários
